# Definitive study shows no association between *ARID1A* mutation status and clinical outcome in endometriosis‐related ovarian cancers‡

**DOI:** 10.1002/path.5973

**Published:** 2022-06-29

**Authors:** Saira Khalique, Sarah Nash, Rachael Natrajan

**Affiliations:** ^1^ The Breast Cancer Now Toby Robins Research Centre, Division of Breast Cancer The Institute of Cancer Research London UK

**Keywords:** ARID1A, CD8 tumour infiltrating lymphocytes, mismatch repair deficiency, biomarker, endometriosis‐associated ovarian carcinomas

## Abstract

The ARID1A tumour suppressor protein is a component of the SWI/SNF chromatin remodelling complex, which is mutated in approximately 20% of all human cancers. *ARID1A* mutational status is considered to hold prognostic significance in a range of solid malignancies, yet in endometriosis‐related ovarian carcinomas there has been a lack of clarity of its prognostic role. Moreover, the relationship between ARID1A status and immune infiltrate is also poorly understood. In a recent issue of *The Journal of Pathology*, a large comprehensive study by Heinze, Nazeran *et al* addressed these areas by reviewing 1,623 endometriosis‐associated ovarian carcinomas and correlating ARID1A status using standardised immunohistochemistry to infer mutation status, with comprehensive clinicopathological features, mismatch repair status and CD8^+^ tumour infiltrating lymphocytes. The study definitively showed that ARID1A status does not provide any independent prognostic value in endometriosis‐associated ovarian carcinomas. ARID1A loss was, however, shown to be associated with mismatch repair deficiency and increased CD8^+^ tumour infiltrating lymphocytes in endometrioid ovarian carcinoma, which may be relevant for future studies. © 2022 The Authors. *The Journal of Pathology* published by John Wiley & Sons Ltd on behalf of The Pathological Society of Great Britain and Ireland.

The high prevalence of *ARID1A* mutations in endometriosis‐associated ovarian cancers (EAOCs), which include endometrioid ovarian carcinoma (ENOC) and clear cell ovarian carcinoma (CCOC), has led researchers to attempt to understand its significance with respect to clinical outcomes and the tumour immune microenvironment. Thus far there has been a lack of clarity on its prognostic role in ovarian carcinomas, with previous reports showing conflicting or lack of association between ARID1A status and clinical outcome. Studies using genetically engineered mouse models have shown that ARID1A deficiency leads to increases in tumour infiltrating lymphocytes (TILs), immune checkpoint activation and subsequent sensitisation to PD‐L1 checkpoint blockade, due to a proposed loss of interaction with the mismatch repair (MMR) protein MSH2 [1]. In patient samples, ARID1A loss has been associated with high CD8^+^ TILs in CCOC, although these studies have been limited by small sample size. To address these areas, Heinze, Nazeran *et al* [2] interrogated large‐scale international tissue collections to definitively correlate *ARID1A* mutational status as measured by protein loss using immunohistochemistry (IHC) with clinical information, MMR status and CD8^+^ TILs.

ARID1A IHC has previously been shown to be a robust biomarker for detecting *ARID1A* loss of function mutations in gynaecological cancers [3]. Heinze and colleagues reviewed more than 5,000 ovarian carcinoma cases, including 1,623 EAOC (1,078 ENOC and 545 CCOC). They report a similar prevalence of ARID1A loss to published studies, with loss of ARID1A staining observed in 25% of ENOC and 42% of CCOCs, with retention of ARID1A protein in more than 95% of both low‐grade (*n* = 111) and high‐grade serous ovarian cancer cases (*n* = 2,548). Importantly, the authors showed that ARID1A loss is not an independent prognostic factor for either ENOC or CCOC when assessing a variety of survival metrics, including overall survival, progression‐free survival and disease‐specific survival. They confirmed, however, the importance of prognostic factors, such as the presence of residual disease and stage, with residual disease and higher stage being associated with poorer outcomes.

A smaller cohort of tumours was assessed for CD8^+^ TILs within the tumour epithelium (933 ENOC and 480 CCOC), with ARID1A loss being associated with statistically significant higher CD8^+^ TILs in ENOC but not in CCOC, although a trend to higher CD8^+^ TILs was seen. A modest yet statistically significant overall survival benefit was observed in ENOC patients with high CD8^+^ TILs scores. No such trend was observed in CCOC. CD8^+^ TILs were significantly associated with MMR deficiency (MMRd) in ENOC and CCOC tumours, with an overall rate of MMRd of 13% of ENOC tumours and 5% of CCOC tumours. A significant association between ARID1A loss and MMRd was noted, present in 22% of all ENOC, highlighting that ARID1A loss is probably confounded by MMRd, given MMRd results in a high mutational burden in an otherwise genomically quiet tumour type.

We would like to commend the authors on this impressive large‐scale international effort highlighting that ARID1A IHC is a reproducible and reliable test for loss of function mutational status, which can be used in mainstream pathology laboratories. Moreover, the authors provide clarity and definitive evidence of the lack of prognostic significance of ARID1A in EAOC. However, these results do not discount the importance of developing therapeutic strategies to target tumours with loss of ARID1A and the clinical relevance of investigating the possible predictive value of ARID1A for immune‐modulatory therapies, given the overall poor clinical outcome in these disease types. One approach utilising synthetic‐lethal approaches for targeting ARID1A‐deficient cancers with the ATR inhibitor ceralasertib with or without the  PARP (poly ADP ribose polymerase) inhibitor olaparib is being assessed in the ATARI phase II international proof‐of‐concept clinical trial. In this trial, ARID1A IHC is being used upfront to stratify patients with ovarian and endometrial clear cell carcinoma, together with other rare gynaecological tumours, into the two treatment arms depending on their ARID1A status.

In this current study, the authors used tissue microarrays as a practical method for obtaining an initial insight into the pathology of each sample. One limitation is that a tissue microarray may fail to accurately capture subclonal staining patterns and intra‐tumoural heterogeneity. In addition, the logistics of sequencing large numbers of patient samples to assess tumour mutational burden and to determine POLE (DNA polymerase ε) status was not possible. Future studies incorporating these elements and serial patient samples would allow a deeper assessment with respect to modern molecular prognostic subtypes of ENOC, tumour evolution and responses to treatment.

Overall, the authors showed that loss of ARID1A is associated with higher CD8^+^ TILs in ENOC and intra‐tumoural CD8^+^ immune cells suggestive of a possible role for immunotherapy. Low response rates have been demonstrated in initial clinical trials in relapsed ovarian cancer. However, CCOC has shown improved response rates compared with other epithelial ovarian cancers, including the highest response rate of 15.8% to pembrolizumab in the phase II KEYNOTE‐100 trial, compared with 8.5% in unselected ovarian cancer. NINJA, a randomised phase III trial in platinum‐resistant ovarian cancer patients, assessed nivolumab compared with chemotherapy, showing no statistically significant survival benefit, but a numerically longer overall survival in CCOC patients (Table 1 highlights ongoing relevant immunotherapy‐based trials). Although Heinze and colleagues show a significant association between ARID1A loss and MMRd, the clinical impact of MMR status in the context of ARID1A loss is not understood and the translational research from the above studies will help to further elucidate the relevance of ARID1A loss depending on MMR status and response to immunotherapy.

**Table 1:** Relevant immunotherapy trials in clear cell gynaecological cancers.

Study title	Phase	Treatment	Primary aims	Secondary aims	Patients (*n*)	Molecular target	Identifier, Status
BrUOG 354 Nivolumab ± ipilimumab for ovarian and extra‐renal clear cell carcinomas	II	Nivolumab ± ipilimumab	PFS	PFS	62	PD‐1 and CTLA4	NCT03355976 Recruiting
Nivolumab and ipilimumab in treating patients with rare tumours	II	Nivolumab and ipilimumab	ORR	AE, BOR, CBR, OS, PFS	707	PD‐1 and CTLA4	NCT02834013 Recruiting
A multicentre phase II trial of durvalumab versus physician's choice chemotherapy in recurrent ovarian clear cell adenocarcinomas (MOCCA)	II	Durvalumab versus standard cytotoxic chemotherapy	PFS	ORR, OS, AE, QOL	46	PD‐L1	NCT03405454 Results awaited
A phase II study of pembrolizumab in patients with advanced gynaecological clear cell cancer (PEACOCC)	II	Pembrolizumab	PFS	QOL	48	PD‐1	NCT03425565 active not recruiting. Results awaited
Phase II trial of lenvatinib plus pembrolizumAb in recurrent gynecological clear cell adenocarcinomas (LARA)	II	Lenvatinib and pembrolizumab	ORR	PFS, DOR	10	RTK (VEGFR1, VEGFR2, VEGFR3) and PD‐1	NCT04699071 Recruiting

AE, adverse event; BOR, best overall response; CBR, clinical benefit rate; DOR, duration of response; OS, overall survival; ORR, overall response rate; PFS, progression‐free survival; QOL, quality of life; RTK, receptor tyrosine kinase.

Although the authors scored CD8^+^ cells, further key immune subpopulations and their spatial locations were not characterised and correlated with *ARID1A* mutational status and clinicopathological features. The CD8^+^ TILs scoring methodology was a semiquantitative approach that only considered CD8^+^ TILs within the epithelial component of the tumour, disregarding stromal CD8^+^ cells [2]. Recently, Khalique *et al* [4] quantified and assessed the spatial locations of various immune subpopulations in 31 microsatellite stable CCOCs using multiplexed immunofluorescence. *ARID1A* mutant cases showed significantly higher CD8^+^ cells and CD68^+^ cells (tumour‐associated macrophages, TAMs) in the stroma relative to tumour. The spatial location of these immune subpopulations has also been shown to hold clinical significance in a range of solid malignancies, such as colorectal, breast and lung cancer [5, 6, 7, 8]. In squamous lung cell cancer, for example, stromal TAMs have been shown to interact with stromal CD8^+^ cells, and this interaction results in a reduction in motility of CD8^+^ cells in the tumour microenvironment, ‘trapping’ them in the stroma and contributing to a T‐cell excluded tumour phenotype associated with worse outcome [9]. This interaction would suggest that targeting TAMs could be relevant in synergising immune checkpoint‐based immunotherapy in various tumour types, and in the context of endometriosis‐related ovarian cancers, specifically *ARID1A‐*mutated tumours. Khalique *et al* [4] also found significantly higher numbers of immunosuppressive subpopulations (TAMs and FOXP3^+^/CD4^+^ regulatory T‐cells) in the stroma relative to tumours of patients with improved outcomes, suggesting that the ‘tumour‐exclusion’ of these cells is important in maintaining an effective anti‐tumour immune response. It would be useful to validate these spatial findings in larger cohorts, such as that of Heinze, Nazeran *et al* [2].

Recent studies have additionally used gene expression profiling to identify prognostic gene expression signatures in CCOCs. Tan *et al* [10] identified two prognostic CCOC gene expression subtypes, including a mesenchymal subtype characterised by a high immune/inflammatory gene expression profile but not driven by CD8^+^‐specific signatures. Khalique *et al* [4] also identified and validated an immune‐related gene expression signature associated with clinical outcome in CCOC. Gene expression analysis in the large cohort of Heinze, Nazeran *et al* [2] would provide valuable additional data to prognosticate these tumours.

This large current study highlights the importance of maintaining clinical databases (although this study was comprised of predominantly Caucasian patients), with international registry collaborations enabling a robust and powerful dataset with which one can draw definitive conclusions. ARID1A has been shown not to be an independent prognostic biomarker and is confounded by MMRd in EAOC. Ongoing research is needed to determine its clinical relevance in additional ethnic populations in a rapidly evolving landscape of treatment options and to take these findings into consideration when interpreting the results of trials where known biomarkers of response include MMRd.

## Author contributions statement

All authors were involved in writing and approving the final version of the manuscript.
